# Directing the osteoblastic and chondrocytic differentiations of mesenchymal stem cells: matrix vs. induction media

**DOI:** 10.1093/rb/rbx008

**Published:** 2017-04-11

**Authors:** Jing He, Jianglong Guo, Bo Jiang, Ruijuan Yao, Yao Wu, Fang Wu

**Affiliations:** * National Engineering Research Center for Biomaterials, Sichuan University, Chengdu 610064, P.R. China

**Keywords:** osteoblastic differentiation, chondrocytic differentiation, induction media, matrix

## Abstract

While both induction culture media and matrix have been reported to regulate the stem cell fate, little is known about which factor plays a more decisive role in directing the MSC differentiation lineage as well as the underlying mechanisms. To this aim, we seeded MSCs on HA-collagen and HA-synthetic hydrogel matrixes, which had demonstrated highly different potentials toward osteoblastic and chondrocytic differentiation lineages, respectively, and cultured them with osteogenic, chondrogenic and normal culture media, respectively. A systematic comparison has been carried out on the effects of induction media and matrix on MSC adhesion, cytoskeleton organization, proliferation, and in particular differentiation into the osteoblastic and chondrocytic lineages. The results demonstrated that the matrix selection had a much more profound effect on directing the differentiation lineage than the induction media did. The strong modulation effect on the transcription activities might be the critical factor contributing to the above observations in our study, where canonical Wnt-β-Catenin signal pathway was directly involved in the matrix-driven osteoblastic differentiation. Such findings not only provide a critical insight on natural cellular events leading to the osteoblastic and chondrocytic differentiations, but also have important implications in biomaterial design for tissue engineering applications.

## Introduction

Mesenchymal stem cells (MSCs) are multipotent cells, present in bone marrow, that are able to differentiate into various anchorage-dependent cell types, including neurons, myoblasts, osteoblasts, chondrocytes and so on [1, 2]. Due to their capacity to differentiate into cells of connective tissue lineages, MSCs are promising candidates for use in cell-based therapies and tissue engineering applications. Directing the MSC differentiation into its desired lineage is critical for tissue regeneration and has drawn continued interests from the researchers. The differentiation lineage of MSC is governed by its chemical and physical environments, including soluble factors [3], chemical and physical cues [4–6], mechanical factors (such as matrix elasticity and tension) [7–9] and the biophysical microenvironment [10].

The usage of induction culture media is a common and effective method to direct the MSC differentiation toward the osteogenic, chondrogenic, adipogenic lineages and so forth [11–15]. For instance, the osteogenic induction medium, containing dexamethasone (Dex), β-glycerophosphate and ascorbic acid, has been generally used to induce the MSC differentiation into osteoblast, by increasing the alkaline phosphatase activity and accelerating bone mineralization [16–18].

While the induction media have been commonly used to direct the MSC differentiation into the desired lineages, such process is sometimes un-natural and is generally only suitable for *invitro* manipulations. A critical component of the stem cell niche is its residing matrix, and numerous studied have reported that the matrix exert a profound effect on various MSC functions, including attachment, proliferation, migration and differentiation [6, 19]. For instance, the selection of the extracellular matrix (ECM) or ECM-derived material has been widely used in modulating and stimulating MSC functions [6, 20]. More interestingly, it has been reported that the matrix itself might exert a critical regulation effect on MSC fate decision. Our previous study revealed that selection of collagen instead of synthesized hydrogel on top of the hydroxyapatite stimulated the MSC differentiation into the osteoblastic lineage, which might be attributed to the accelerated MSC condensation and robust cell-matrix and cell-cell interactions [21]. Indeed, the ECM provides a favorable microenvironment for various cellular responses, such as survival, migration and differentiation, by promoting adhesion, modulating the activities of growth factors and cytokines and directly activating intracellular signaling [21]. Besides, surface function groups also exert strong influence on the stem cell lineage specification, though modulating the structure and molecular composition of cell-matrix adhesions [22–25]. In addition, the matrix varies not only in chemical composition, but also in physical properties, including the elasticity and topography. Such physical properties would also influence a range of cellular processes through changes in focal adhesion and the actin cytoskeleton [26, 27]. For example, Engler *et al.* showed that matrix elasticity can specify stem cell lineage toward neurons, myoblasts and osteoblasts, indicating a critical role of physical environment on MSC fate decisions [28].

In short, previous studies have shown that both the induction culture media and matrix could regulate stem cell fate. However, which factor plays a more decisive role in directing the MSC differentiation lineage, and the underlying mechanisms for both regulating effect remain to be elucidated. For instance, the osteogenic-based HA-collagen matrix seems have a strong influence on the transcriptional factor Runx2, but would that be the main mechanism and how would the matrix affect the transcriptional activities? On the other hand, whether the osteogenic induction media (Dex) affect the transcriptional activity or not remains controversial [11]. It is also of great interest to understand the potential correlating role of both factors for clinical applications in bone and cartilage repair.

To address the above questions is of great importance for tissue engineering applications. For the treatment of osteochondral damage, it is desirable to design new biomaterial scaffolds that provide different and adequate conditions for guiding the growth of both cartilage and subchondral bone, satisfying their different biological and functional requirements [29, 30]. Different approaches have been adopted for bone and cartilage regenerations, by using the induction media and matrix selection, or the combination of both. For example, Mikos have used the same injectable and biodegradable hydrogel to fabricate a bi-layered osteochondral construct by using different growth factors or induction media to promote the MSC differentiation into the respective lineages [31]. Chen and co-workers have designed a novel layered osteochondral scaffold consisting of the upper collagen layer and the lower collagen/HA layer to simultaneously drive the chondrogenic and osteogenic differentiations, respectively [32]. Nevertheless, they were insufficient in driving the lineage commitment and still required induction media to accelerate the osteogenesis [31, 32]. It is important to understand the underlying mechanisms of how both factors affect the osteogenesis and chondrogenesis.

The current study aimed to address the above question by performing a parallel comparison between induction media and matrix in terms of their capabilities to modulate the MSC fate into the osteoblastic and chondrocytic lineage. We selected two matrixes: HA-collagen and HA-synthetic hydrogel, which had showed highly different potentials toward osteoblastic and chondrocytic differentiation lineages, respectively [21]. The MSCs were seeded on the above two matrix surfaces, and cultured under three different culture media, i.e. the normal culture medium, osteogenic induction medium, and chondrogenic induction medium, respectively. The effects of both matrix and culture media on the osteoblastic and chondrocytic differentiation of MSCs were systematically compared. In particular, the effects of these two factors on the transcriptional activities were thoroughly analysed and the underlying mechanisms were vigorously exploited. We further analysed the role of Wnt signaling pathway in the regulation effect of matrix on the osteoblastic transcription activities, which plays a critical role in mechanotransduction and in activating osteoblastic differentiation.

## Materials and methods

### Materials preparation

#### Preparation of the HA-collagen coating and the HA-synthetic hydrogel coating.

Porous HA coating was synthesized as previously reported [33]. To prepare the HA-collagen coating, 400 μl collagen solution (7 mg/ml, pH 5.0) containing 12.5 μg rhBMP-2 was dropped on the HA coating surface using a previous described method [34]. The HA-synthetic hydrogel (PLGA-PEG-PLGA) coating was prepared in the same way as the preparation method for HA-collagen matrix [21]. In brief, each HA coating sample was added by 400 μl PLGA-PEG-PLGA hydrogel solution with additional incorporation of 12.5 μg rhBMP-2. The BMP was added to promote the MSC differentiation, and it was reported that BMP stimulated both the osteoblastic and chondrocytic differentiations of MSCs [21].

#### Preparation of culture media.

Osteogenic induction media was prepared, consisting of DMEM supplemented with 10% fetal bovine serum (FBS), 100 nM dexamethasone, 10 mM sodium β-glycerolphosphate, and 0.05 mM 1-ascorbic acid-2-phosphate, a well-known formulation for differentiation media [35].

Chondrogenic induction media was purchased from Cyagen biosciences Inc., consisting of serum-free DMEM supplemented with dexamethasone, ascorbate, ITS + supplement, sodium pyruvate, proline and TGF-β3.

Normal culture media was prepared, containing α-MEM supplemented with 20% FBS.

### MSC harvesting and culture

Isolation and culture of MSCs were performed as previously described [34]. MSCs were obtained from the femora and tibias of 3-days-old New Zealand rabbits. MSCs were grown in α-MEM containing 20% FBS and 1% antibiotics (HyClone, Thermo Fisher Scientific Inc., USA), and cells from passage 3 were used for all experiments.

### Cell proliferation and morphology

Suspension of MSCs with number 2 × 10^4^ per milliliter was seeded on HA-collagen and HA-synthetic hydrogel substrate surfaces, and was cultured with the osteogenic induction media, chondrogenic induction media and normal culture media, respectively. The proliferation of MSCs on the samples was measured by cell counting kit-8 (CCK-8) assay after culture 2, 4, 6 and 14 days. Confocal microscopy was used to analyse the cell morphology on the sample surfaces, after 4 days of culture.

For the following cellular response study, the MSCs were cultured on the HA-collagen and HA-synthetic hydrogel substrates, with osteogenic induction media, chondrogenic induction media and normal culture media, respectively.

### ALP staining

The ALP activity was assayed using a BCIP/NBT Alkaline Phosphatase Color Development Kit (Beyotime, Haimen, China), after 2 weeks of culture. All the experiments were conducted strictly according to the instructions of the BCIP/NBT Alkaline Phosphatase Color Development kits.

### Immunofluorescence staining

After the MSCs being cultured for 6 days, the cells were fixed for 15 min in a 3.7% formaldehyde solution. Cell membranes were permeated by soaking with 0.5% Triton-X100 solubilizing agent for 15 min. Cell were stained with collagen II antibodies: anti-collagen II antibody (1:50 dilution in 0.1%BSA/PBS; Mouse anti-collagen II; Bioss, China), secondary collagen II antibody (1:100 dilution in 0.1%BSA/PBS; FITC conjugated goat anti-mouse Ig G; Bioss, China), incubating at 37 ˚C during each step and rinsing with PBS solution between each step.

### Enzyme-linked immunosorbent assay

The quantitative enzyme-linked immunosorbent assay (ELISA) was used to evaluate the activity of the alkaline phosphates (ALP), and the expressions of runt-related transcription factor-2 (Runx2), osteocalcin (OCN), Osterix, collagen type II (Col II), collagen type X (Col X), N-Cadherin, Wnt-1,Wnt-3a, axin and β-Catenin. All the experiments were conducted strictly according to the instructions of the ELISA kits (BlueGene, Bluegene Ltd., Shanghai, China). The colorimetric products were analysed at 450 nm, and readings were compared with a standard curve.

### Statistical analysis

Results were expressed as mean values ± standard deviation for all experiments. Differences between experimental groups were assessed by one-way analysis of variance (ANOVA) with Bonferoni’s method. Probability value (*P*) <0.05 was considered to be statistically significant.

## Results

### MSC differentiation

#### Osteoblastic differentiation.

**Figure 1:**
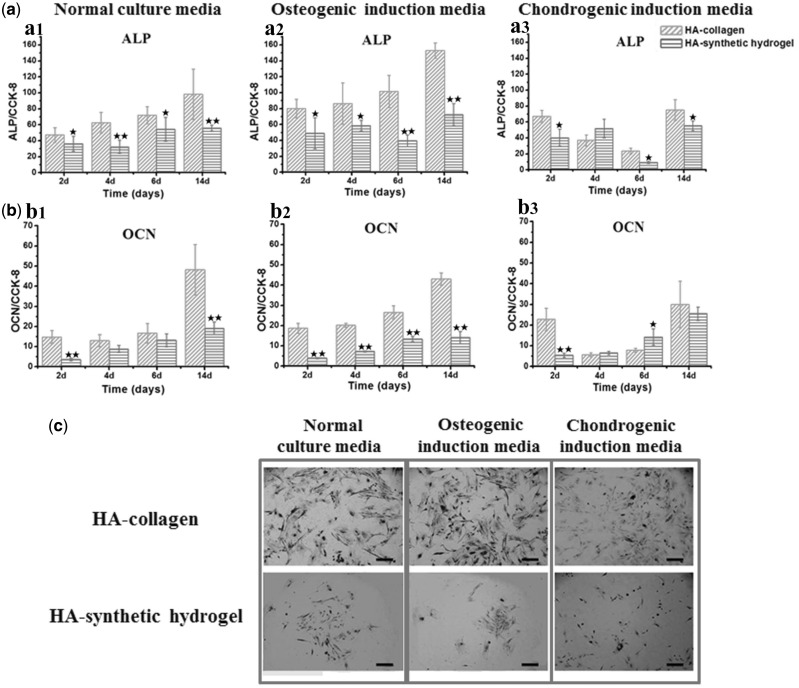
Osteoblastic differentiation. The ALP activities (**a**) and OCN secretions (**b**) of MSCs on the HA-collagen and HA-synthetic hydrogel substrates cultured with normal culture media, osteogenic induction media and chondrogenic induction media for 2, 4, 6, 14 days, respectively. a1 and b1: normal culture media; a2 and b2: osteogenic induction media; a3 and b3: chondrogenic induction media. Error bar represent means ± standard error of mean for *n* = 3. (*P < 0.05 as compared to HA-collagen samples; **P < 0.01 as compared to HA-collagen samples). **c**: ALP staining of MSCs on different sample surfaces

To analyse the osteogenic differentiation of MSCs, the intracellular ALP activity and OCN secretion (Fig. 1) were measured. Under the normal and osteogenic culture conditions, the ALP activities of the MSCs on the HA-collagen substrates were significantly higher than those of the HA-synthetic hydrogel substrates for all times (Fig. 1a1, a2). The ALP activities were significantly suppressed under chondrogenic induction media for both matrices (Fig. 1a3). Similar trend has been found on the OCN secretions. The HA-collagen samples had significantly higher OCN secretions than the HA-synthetic hydrogel samples did with the normal and osteogenic culture medium (Fig. 1b1, b2). The selection of the matrix (HA-collagen vs. HA-synthetic hydrogel) had a profound effect on the osteogenic activities despite of the culture medium used. The selection of the culture media also exerted certain effect on ALP activities for both matrixes, but only affected the OCN secretion with HA-collagen matrix. For HA-synthetic hydrogel, the OCN secretions kept flat regardless of the culture media used.

The ALP staining also confirmed the striking modulating effect of matrix on osteoblastic differentiation, showing significant higher staining for HA-collagen compared with the HA-synthetic hydrogel, especially under the normal and osteogenic culture conditions (Fig. 1c). After incubation for 2 weeks, nodular ALP positive areas were clearly visible on HA-collagen substrates for all culture media, with the biggest area under the osteogenic culture condition. In contrast, almost no discernible mineralized nodule was found on the HA-synthetic hydrogel substrates regardless of the culture media used (Fig. 1c).

#### Chondrocytic differentiation.

**Figure 2:**
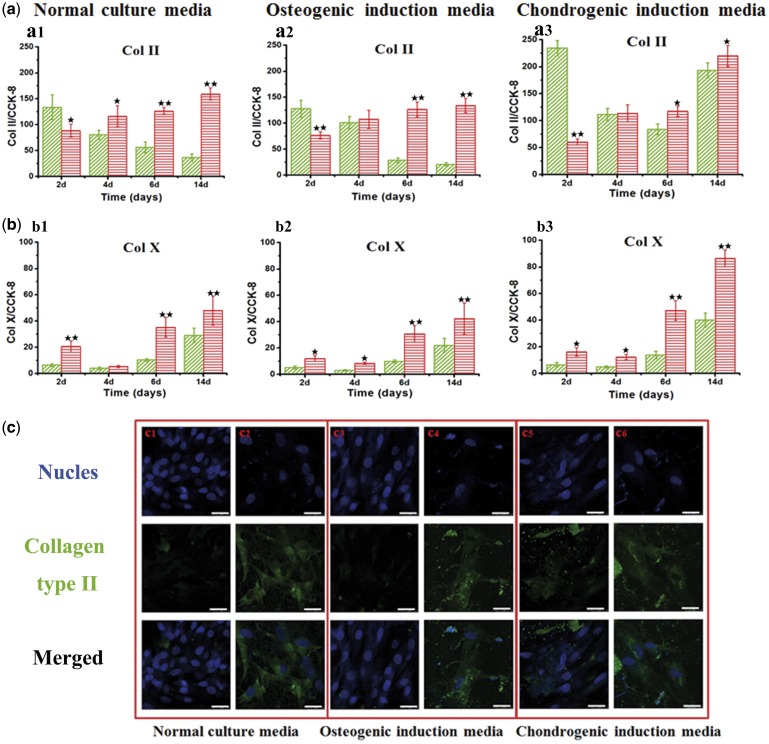
Chondrocytic differentiation. The Col II secretions (**a**) and Col X secretions (**b**) of MSCs on the HA-collagen and HA-synthetic hydrogel substrates cultured with normal culture media, osteogenic induction media and chondrogenic induction media for 2, 4, 6, 14 days, respectively. a1 and b1: normal culture media; a2 and b2: osteogenic induction media; a3 and b3: chondrogenic induction media. Error bar represent means ± standard error of mean for *n* = 3. (*P < 0.05 as compared to HA-collagen samples; **P < 0.01 as compared to HA-collagen samples). **c**: Immunofluorscence staining of collagen type II from MSCs on different sample surfaces. c1: MSCs on the HA-collagen with normal culture media; c2: MSCs on the HA-synthetic hydrogel with normal culture media; c3: MSCs on HA-collagen with osteogenic induction media; c4: MSCs on HA-synthetic hydrogel with osteogenic induction media; c5: MSCs on HA-collagen with chondrogenic induction media; c6: MSCs on HA-synthetic hydrogel with chondrogenic induction media. Blue (nucleus); green (collagen type II)

In order to evaluate the chondrogenic potentials, both the type II and type X collagens were selected as the markers, indicating the early and latter chondrogenic differentiation, respectively (Fig. 2). Regardless of the culture media used, the Col II secretion levels on the group of HA-collagen substrates decreased as a function of cell culture time except for a more moderate decrease with chondrogenic induction media, in contrast to the observed steady increase for the group of HA-synthetic hydrogel substrates (Fig. 2a). At 6 and 14 days, the Col II secretions of the HA-synthetic hydrogel substrates were significantly higher than those of the HA-collagen substrates in the normal and osteogenic culture media. As for the Col X secretions, the regulating effect of matrix was also prominent and the HA-synthetic hydrogel substrates had significantly higher Col X secretions compared with the HA-collagen substrates for all three different culture media (Fig. 2b).

We then performed immunofluorescent staining for specific antibodies for type II collagen, after 14 days of culturing. Collagen type II, known as a major specific cartilage marker, is highly expressed during the MSC differentiation into the chondrocytic lineage. Under the normal and osteogenic culture media, almost no collagen II was detected on the HA-collagen substrates, whereas there was an appreciable secretion of collagen II matrix component on the HA-synthetic hydrogel substrates (Fig. 2c). Under the chondrogenic culture media, collagen II positive areas were found on the HA-collagen samples but were significantly smaller than those on the HA-synthetic hydrogel samples.

### Cell-matrix interactions and cell-cell interactions

**Figure 3:**
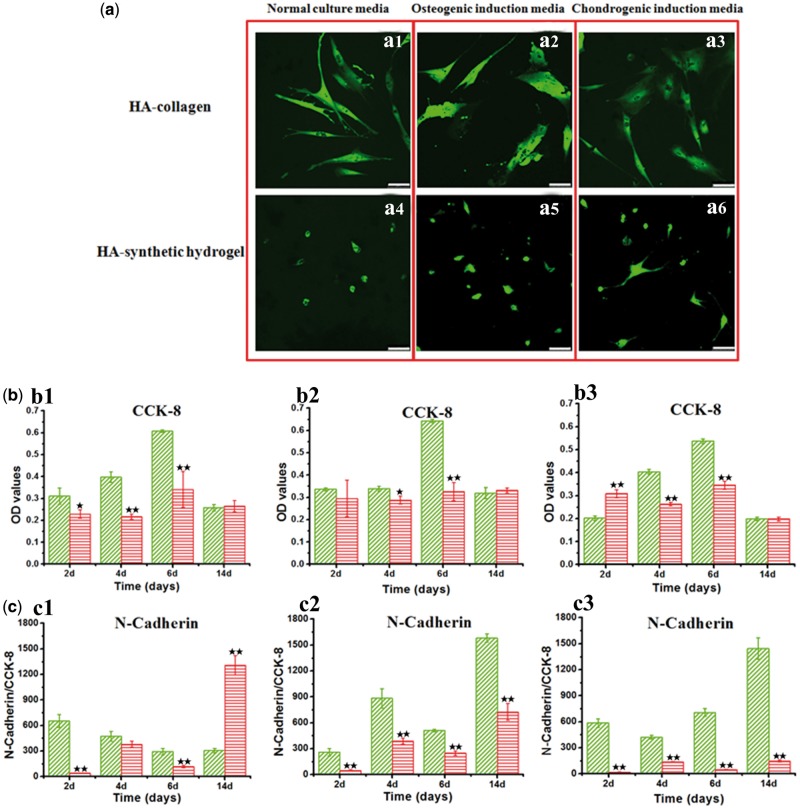
Cell-matrix interactions and cell-cell interactions. **a**: Confocal microscopy of MSCs cultured on different samples with FDA dye for cytoplasm after 4 days. MSCs on the HA-collagen substrates with the normal culture media (a1), osteogenic induction media (a2) and chondrogenic induction media (a3); MSCs on the HA-synthetic hydrogel with the normal culture media (a4), osteogenic induction media (a5) and chondrogenic induction media (a6). **b**: CCK-8 assay for proliferation of MSCs cultured on the HA-collagen and HA-synthetic hydrogel substrates for 2, 4, 6,14 days. Error bar represent means ± standard error of mean for *n* = 3. (*P < 0.05 as compared to HA-collagen samples; **P < 0.01 as compared to HA-collagen samples). **c**: The expression of N-Cadherin of MSCs on the HA-collagen and HA-synthetic hydrogel substrates cultured with the normal culture media (c1), osteogenic induction media (c2) and chondrogenic induction media (c3) for 2, 4, 6, 14 days, respectively. Error bar represent means ± standard error of mean for *n* = 3. (*P < 0.05 as compared to HA-collagen samples; **P < 0.01 as compared to HA-collagen samples)

The fundamental cellular processes would likely affect the cell-matrix and cell-cell interactions and exert certain influence on MSC differentiation. We first examined the adhesion and proliferation of the MSCs on the different matrix surfaces in the different culture media, respectively (Fig. 3). MSCs adhered on all surfaces, but significant differences in cell morphology were observed between the HA-collagen and HA-synthetic hydrogel substrate surfaces (Fig. 3a). For all three culture media, the cells attached well on the HA-collagen sample surfaces (Fig. 3a1–3). In contrast, fewer cells were observed on the HA-synthetic hydrogel sample surfaces and with poorer attachment and extending than that on the HA-collagen coating (Fig. 3a4–6). It is noted that the selection of culture media had minimal effect on the cell morphology and adhesion for both matrices. Data from the CCK-8 assay also show that the number of cells on the HA-collagen sample surfaces was higher than that on the HA-synthetic hydrogel ones, especially at the 6 days (Fig. 3b). Furthermore, the selection of the culture media did not change the overall trends of the MSC proliferation on HA-collagen and HA-synthetic hydrogel substrates. Overall, the results indicated that the selection of matrix played a much critical role in the cell morphology and proliferation than the culture media did.

**Figure 4:**
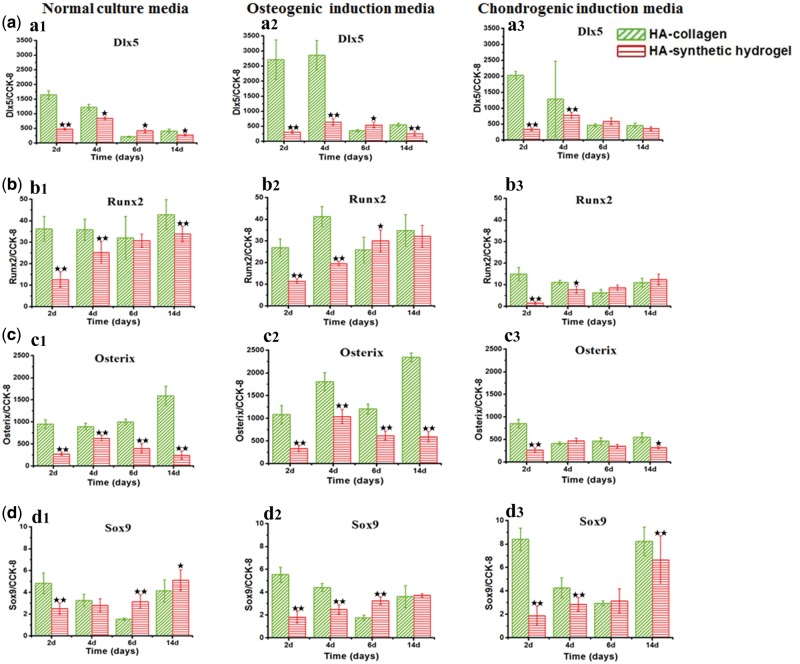
Transcription factors for osteoblastic differentiation. Transcription factors related protein expressions of MSCs cultured on HA-collagen and HA-synthetic hydrogel sample surfaces with normal culture media, osteogenic induction media and chondrogenic induction media for 2, 4, 6, 14 days, respectively. **a**: Dlx5; **b**: Runx2; **c**: Osterix; **d**: Sox9. Error bar represent means ± standard error of mean for *n* = 3. (*P < 0.05 as compared to HA-collagen samples; **P < 0.01 as compared to HA-collagen samples)

In addition, ELISA assay was used to quantify the expressions of N-Cadherin, which is the key molecule involved with cell adhesion and cell-cell interaction [36, 37]. In the normal culture media, the expressions of N-Cadherin on the HA-collagen samples were significantly higher than these of the HA-synthetic hydrogel samples at 2, 4 and 6 days, but were significantly lower at 14 days (Fig. 3c). In the presence of either osteogenic or chondrogenic induction media, significantly higher expressions of N-Cadherin were found on the HA-collagen samples than the HA-synthetic hydrogel samples throughout the culture time (Fig. 3c).

### Molecular mechanisms

#### Transcription factors for osteoblastic differentiation.

To elucidate the underlying molecular mechanisms of the regulating effect of residing matrix and culture media on MSC differentiation, we have examined the transcription activities leading to the osteoblastic differentiation (Dlx5, Runx2 and Osterix [38]) and chondrocytic differentiations (Sox9), respectively. Runx2 is a critical transcription factor for osteoblast differentiation and chondrocytic maturation, whereas Osterix provides specificity in osteoblastic differentiation [38]. Dlx5 is a homeobox domain transcription factor that mediates the expression of Runx2 and Osterix and also regulates osteogenesis [39]. The Runx2 activities of MSCs on the HA-collagen samples were significantly higher than those on the HA synthetic hydrogel ones (Fig. 4b), except for a later stage increase in HA-synthetic hydrogel samples which was likely related to the involvement of Runx2 with chondrocytic maturation besides its role in osteoblastic differentiation. For both substrates, the osteogenic induction media had little effect on Runx2, in contrast to a significant inhibition under chondrogenic media. More profound regulating effect of matrix had been found on the Osterix secretions throughout the culture periods (Fig. 4c) and Dlx5 secretions at early culture times (Fig. 4a), with drastic increases observed for the HA-collagen substrates over the HA-synthetic hydrogel ones regardless the culture medium used.

**Figure 5:**
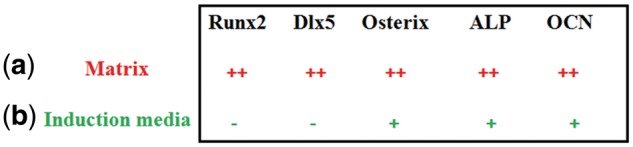
The relative effect of matrix (**a**) and induction media (**b**) on transcriptional factors and differentiation markers

Overall the results indicated an overwhelming effect of residing substrate on all three transcription factors leading to the osteoblastic differentiation: Runx2, Osterix, Dlx5. The culture media however showed a relatively minor influence, and such influence was also matrix-dependant. For HA-synthetic hydrogel substrate, the culture media had almost no influence on all three markers except for showing a weak inhibitory effect on the Runx2 under the chondrocytic induction media (Fig. 4b3). For HA-collagen substrate, the culture media had more profound influence, with more drastic increase of Osterix and Dlx5 observed under the osteogenic induction media. This indicated a likely synergistic effect of matrix and induction media on promoting the Osterix and Dlx5 transcription activities, whereas little synergy was observed on ALP activities (Fig. 5).

Next, we examined the expression of Sox9 which is the most critical transcription factor regulating the MSC condensation and differentiation into the chondrocyte [40]. For all three culture media, the Sox9 expression on the HA-collagen group samples overall decreased as a function of culture time except for keeping at a more steady level in the chondrogenic induction media while an upward trend was found for the HA-synthetic hydrogel group samples (Fig. 4d). Overall, a weak effect of the culture media on Sox9 secretions was observed.

#### *Wnt*-β-*catenin signal pathway*.

**Figure 6:**
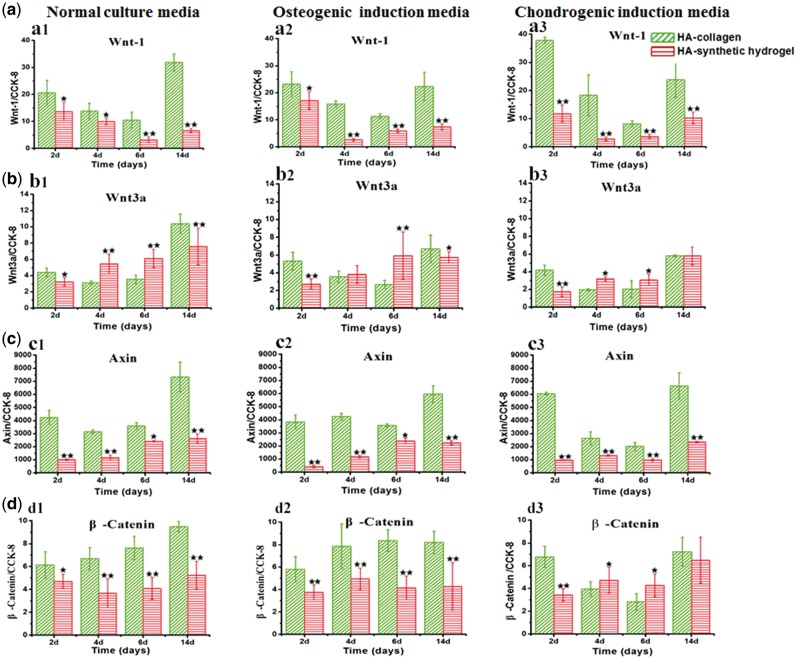
Wnt-β-Catenin signal pathway. Wnt-β-Catenin signal pathway related protein expressions of MSCs cultured on HA-collagen and HA-synthetic hydrogel substrates with normal culture media, osteogenic induction media and chondrogenic induction media for 2, 4, 6, 14 days, respectively. **a**: Wnt1; **b**: Wnt3a; **c**: Axin; **d**: β-Catenin. Error bar represent means ± standard error of mean for *n* = 3. (*P < 0.05 as compared to HA-collagen samples; **P < 0.01 as compared to HA-collagen samples)

To understand the observed significant difference in the transcription activities leading to the osteoblastic differentiation, we further examined the key markers (Wnt-1, Wnt-3a, axin, β-Catenin) in the Wnt-β-Catenin signal pathway, which plays important roles in both mechanotransduction and commitment of osteoblastic differentiation. The results also showed a dramatic regulation effect of matrix itself on the activation of the Wnt-β-Catenin signal pathway, in contrast to a much weaker dependence on the induction media (Fig. 6). Overall, the Wnt-1, axin and β-Catenin expressions of MSCs on HA-collagen substrates were significantly higher than those on the HA-synthetic hydrogel ones, regardless of the selection of the culture medium. On the other hand, the selection of the culture media had much less effect on the above three markers, especially for the HA-synthetic hydrogel matrix. The Wnt-3a, however had a much less dependence on both the matrix and the culture condition. These results suggest that the selection of the proper matrix itself had a vital role in activating the Wnt-β-Catenin signal pathway.

## Discussion

Different approaches have been used to directing the MSC differentiation into the desired lineage, which is critical in tissue engineering applications [5, 7, 10, 41]. While attempts have been made to design biomaterials that might differentiate specifically into either osteogenic or chondrogenic lineage, most ‘bioactive’ materials selected generally improve the proliferation and promote the differentiation into both lineages without a clear preference. For bone and cartilage tissue engineering applications such as osteochondral constructs, osteogenic and chondrogenic induction medium have been commonly used to initiate the osteoblastic and chondrocytic differentiations, respectively. We have recently reported that the matrix itself had a vital effect on directing the MSC differentiation lineage and the selection of the bone formation mode [21]. A simple combination of the hydroxyapatite (HA) with collagen stimulates the MSC differentiation into the osteoblastic lineage and intramembraneous bone formation mode *invivo*, whereas the HA-synthetic hydrogel matrix promotes the MSC differentiation into the chondrocytic lineage followed by the endochondrol bone formation mode [21]. In this study, a systematic comparison of the effects of induction media and the matrix on the osteoblastic and chondrocytic differentiations and the corresponding molecular mechanisms have been carried out. The results demonstrated that the selection of the matrix (HA-collagen or HA-synthetic hydrogel) had a much more profound effect on directing the differentiation lineage than the induction media did.

For osteoblastic differentiation, the selection of HA-collagen matrix over the HA-synthetic hydrogel significantly increased the ALP and OCN expressions regardless of the culture condition, whereas the osteogenic induction media only moderately increased the ALP activity and showed no effect on the OCN secretion (Fig. 1). On the other hand, the HA-synthetic hydrogel matrix strongly favored the chondrocytic differentiation regardless of the culture conditions, showing an upward trend for Col II and significantly up-regulation of Col X under all three culture media. The chondrogenic induction media however had almost no effect on Col II secretion and moderately increased the Col X secretion for HA-collagen matrix alone. The results clearly suggested a striking role of matrix/substrate in directing the MSC differentiation lineage (Fig. 2).

Then we wonder why matrix has a considerably larger role in dictating the MSC differentiation lineage by first examining the effect of matrix and induction media on various MSC functions. The MSCs had much superior cell adhesion and cell proliferation on the HA-collagen substrate compared with the HA-synthetic hydrogel substrate, whereas the presence of induction medium did not change the results of cell adhesion and proliferation. The observed significantly higher expression of N-Cadherin, which is related to the cell-cell interactions, was a strong indication of robust cell-cell interactions among the MSCs on HA-collagen matrix. Presence of induction culture media led to the significant N-Cadherin up-regulation for HA-collagen substrate (Fig. 3).

Then we examine the effect of induction media and matrix on the transcriptional activities. The osteoblastic transcription factors Runx2, Osterix and Dlx5 regulate osteoprogenitor differentiation, and their activities are intricately connected. The HA-collagen matrix had very significantly higher Osterix and Dlx5 expression levels than the HA-synthetic hydrogel group samples did, whereas the induction media showed little effect on these two expressions except for a minor inhibition on Osterix under chondrogenic condition. The increase of Runx2 expression by using the HA-collagen over HA-synthetic hydrogel substrate was not as substantial as the former two. This was likely due to the dual role of Runx2 in both osteogenesis and chondrogenesis whereas the other two are mainly osteogenic factors. Runx2 not only control osteoblast differentiation but also participate in chondrocytic maturation [38]. This was consistent with the gradual increase of Runx2 over the time for the HA-synthetic hydrogel substrates, showing strong tendency toward chondrocytic differentiation. Less influence had been found on the chondrogenesis markers Sox9 from the matrix, despite of the observed increased Sox9 levels on the HA-synthetic hydrogel matrix. Besides its role in chondrocytic differentiation, Sox9 is also critical in MSC condensation, prior to the MSC commitment to either osteoblastic or chondrocytic lineage [40].

The relative effect of matrix and induction media on differentiation markers and transcriptional factors are listed in Fig. 5, respectively. Overall, the results indicated that the matrix had a strong modulation effect on the transcription activities (in particular on the osteoblastic differentiation), leading to dramatic increase in Runx2, Osterix and Dlx5 expressions (Fig. 5a). The induction media however exerted little effect on the transcriptional activities, and only weakly affected the Osterix expressions (Fig. 5b).

Then we further exploited the likely mechanisms governing the strong modulation effect of matrix on the transcriptional activities. There are two possible mechanisms by which the matrix modulates the transcriptional activities. Firstly, the integrins on the ECM binding to the cell surface receptors could active the specific chemical signal, and the integrating signals from the ECM would likely affect a cascade of events and provide signaling platforms for modulating gene transcription involved in cell adhesion, migration, proliferation and differentiation [42]. Nevertheless, the increased cell adhesion and proliferation found on the HA-collagen substrate might not be the decisive factor leading to the commitment of osteoblastic differentiation. The HA-collagen matrix promoted both osteoblastic and chondrocytic differentiation at early stage (3 day), as indicated by the enhanced ALP activity and Col II secretions on HA-collagen sample at 3 days. However, the selection of the ECM protein as the matrix would facilitate the cell-matrix interactions and cell-cell interactions, which might have an indirect role in affecting the transcriptional activities. The expression of N-Cadherin, a calcium-dependent cell adhesion molecule that mediates the cell-cell interaction [43], was significantly up-regulated by HA-collagen matrix at the early period (3, 5 and 7 days). The N-Cadherin thus might be an important factor contributing to the commitment of osteoblastic lineage. It is well documented that N-Cadherin is critical in the maintenance of osteoblastic phenotype, and use of N-Cadherin function blocking antibodies impairs the osteoblastic differentiation [44–47]. Our previous studies also suggested accelerated cell-cell interactions might be critical for the selection of osteogenic differentiation instead of chondrogenic differentiation [21]. Again, the MSCs exhibited the highest expressions of N-Cadherin on HA-collagen substrate, in consistence with the highest osteoblastic differentiation activities observed on HA-collagen substrate.

The other more likely mechanism accounting for the modulatory effect of matrix on the transcriptional activities is mechanotransduction, which is vital in osteogenesis [48]. The fibrous structure of the collagen matrix provides a critical microenvironment for MSC adhesion and migration, which is important for MSCs to sense and respond to the stress cues [6, 49]. The underlying HA substrate, not only provides a suitable stiffness for MSC functions, but also facilitates the collagen self-organization and formation of the fibrous network [50]. We have examined the Wnt-1, Wnt-3a, axin, β-Catenin, four key markers in the canonical Wnt-β-Catenin signal pathway, which plays a critical role in mechnaotransduction and in activation of the osteoblastic differentiation. The results showed that the HA-collagen matrix had strong stimulation effect on the activation of the Wnt-β-Catenin signal pathway, by significantly up-regulating the expressions of Wnt-1, axin, β-Catenin, where only minor influence was observed from the induction media. The observed activation of Wnt-β-Catenin signal pathway on HA-collagen matrix strongly correlated the significantly up-regulated transcriptional activities (Runx2, Osterix and Dlx5) toward the osteogenic lineage (Fig. 6).

The results suggested a HA-collagen matrix-driven osteoblastic differentiation for MSCs, likely through a series of coordinated cellular events. The collagen-based materials have been reported to promote both the osteoblastic and chondrocytic differentiations [51, 52]. Nevertheless, the HA-collagen matrix selected in this study, while having a structure far from the ideal hierarchical structure from the natural bone, did imitate the natural composition of bone in a simple manner and likely provided the proper physical microenvironment and chemical microenvironment favoring the osteoblastic differentiation, a highly robust process as occurred in natural bone development and regeneration. Less was understood for the observation that the HA-synthetic hydrogel matrix stimulated the chondrocytic differentiation, as it imitated neither the composition nor the structure of the natural cartilage. One possible explanation is that the chondrocytic differentiation is preferred when there is an arrest of osteoblastic differentiation. Since significant Col X up-regulations have been observed for the HA-synthetic hydrogel substrates, it is desirable to exploit new matrix candidate that would stimulate chondrocytic differentiation and suppress the chondrocyte hypertrophy simultaneously.

**Figure 7:**
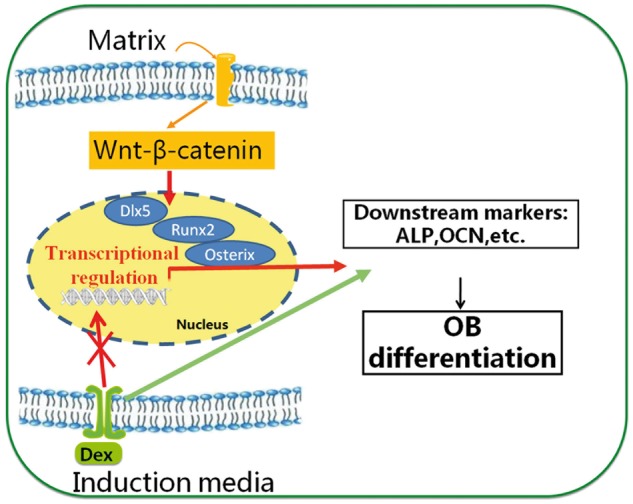
Road maps of induction media and matrix directed MSC differentiation

Overall, our results demonstrated that the residing matrix/substrate had a profound effect on various MSC functions including adhesion, proliferation, migration and differentiation. While the selection of the HA-collagen substrate over the HA-synthetic hydrogel greatly enhanced the MSC adhesion and proliferation, it unlikely dictated the MSC differentiation into the osteoblastic lineage. However, the N-Cadherin expression, a critical indication for cell-cell communication, might be an important factor contributing to the commitment to the osteoblastic lineage. Nevertheless, the fundamental difference between the underlying mechanisms of how the matrix and induction media modulate the MSC differentiation lies in the overwhelmingly driving influence of the matrix on the transcriptional activities leading to the respective differentiation lineages, especially for the osteoblastic differentiation. This is in contrast to a minimal effect from the induction media, as shown schematically in Fig. 7. The canonical Wnt-β-Catenin signal pathway might be critically involved with the matrix driven osteoblastic differentiation. Our results clear demonstrated that the selection of the proper matrix was more decisive in driving the lineage commitment than the induction media did, at least in our selected systems. Such findings not only provide a critical insight on understanding the natural cellular events that lead to the osteoblastic and chondrocytic differentiations, but also have important implications in biomaterials design in tissue engineering applications, such as osteochondral constructs.

## Conclusions

Our present study showed that compared with the induction media, the selection of the matrix (HA-collagen vs. HA-synthetic hydrogel) had a more profound effect on MSC adhesion, cytoskeleton adhesion, proliferation, and differentiation into the osteoblastic and chondrocytic lineages. The selection of the HA-collagen substrate drove the MSC differentiation into the osteoblastic lineage, and in particular through its strong modulation effect on the transcription activities leading to the osteoblastic differentiation, as evidenced by the strong up-regulation of Runx2, Osterix and Dlx5 expressions. In contrast, the induction media exerted little effect on the transcriptional activities, and only weakly affected the Osterix expressions. The canonical Wnt-β-Catenin signal pathway might be critically involved with the matrix driven osteoblastic differentiation. Overall, our results clear demonstrated that the selection of the matrix had a much more critical effect on directing the differentiation lineage than the induction media did.
